# Feasibility and acceptability of regular weighing, setting weight gain limits and providing feedback by community midwives to prevent excess weight gain during pregnancy: randomised controlled trial and qualitative study

**DOI:** 10.1186/s40608-015-0061-5

**Published:** 2015-09-16

**Authors:** AJ Daley, K. Jolly, SA Jebb, AL Lewis, S. Clifford, AK Roalfe, S. Kenyon, P. Aveyard

**Affiliations:** Primary Care Clinical Sciences, School of Health and Population Sciences, College of Medical and Dental Sciences, University of Birmingham, Birmingham, B15 2TT West Midlands; Public Health, Epidemiology and Biostatistics, School of Health and Population Sciences, University of Birmingham, Birmingham, B15 2TT West Midlands; Nuffield Department of Primary Care Health Sciences, University of Oxford, Radcliffe Observatory Quarter, Woodstock Road, Oxford, OX2 6GG UK; School of Social and Community Medicine, University of Bristol, Canynge Hall, 39 Whatley Road, Bristol, BS8 2PS South West England

**Keywords:** Gestational weight gain, Regular weighing, Weight gain limits, Community midwives

## Abstract

**Background:**

Regular weighing in pregnant women is not currently recommended in many countries but has been suggested to prevent excessive gestational weight gain. This study aimed to establish the feasibility and acceptability of incorporating regular weighing, setting maximum weight gain targets and feedback by community midwives.

**Methods:**

Low risk pregnant women cared for by eight community midwives were randomised to usual care or usual care plus the intervention at 10–14 weeks of pregnancy. The intervention involved community midwives weighing and plotting weight on a weight gain chart, setting weight gain limit targets, giving brief feedback at each antenatal appointment and encouraging women to weigh themselves weekly between antenatal appointments. Women and midwives were interviewed about their views of the intervention. The focus of the study was on process evaluation.

**Results:**

Community midwives referred 123 women and 115 were scheduled for their dating scan within the study period. Of these, 84/115 were approached at their dating scan and 76/84 (90.5 %) randomised. Data showed a modest difference favouring the intervention group in the percentage of women gaining excessive gestational weight (23.5 % versus 29.4 %). The intervention group consistently reported smaller increases in depression and anxiety scores throughout pregnancy compared with usual care. Most women commented the intervention was useful in encouraging them to think about their weight and believed it should be part of routine antenatal care. Community midwives felt the intervention could be implemented within routine care without adding substantially to consultation length, thus not perceived as adding substantially to their workload.

**Conclusions:**

The intervention was feasible and acceptable to pregnant women and community midwives and was readily implemented in routine care.

**Trial registration:**

ISRCTN81605162

## Background

Average weight gain in pregnancy has increased in the last two decades across the full range of pre pregnancy body mass index categories with more than 60 % of pregnant women exceeding current US Institute of Medicine (IOM) guidelines [1–7]. Excess gestational weight gain is associated with postnatal weight retention up to 10–15 years after pregnancy in all BMI categories of women [8–12]. The weight women gain during pregnancy but fail to lose after pregnancy leads to incremental gain across successive pregnancies [13]. In addition, studies report an association between high maternal weight gain during pregnancy and increased adiposity and morbidity in children [14, 15]. Excessive gestational weight gain is also associated with several adverse outcomes such as gestational diabetes, pre-eclampsia, delivery complications, macrosomia and still birth that could be prevented with effective weight control interventions [1, 16–18].

Women often report that gaining weight during pregnancy is inevitable and weight management is less important when pregnant and consequently reduce their physical activity and consume a more liberal diet [19–22]. There have been calls for weight management to be integrated into routine antenatal care but there is a paucity of evidence of effective interventions to prevent excessive gestational weight gain [23]. Community midwives are the ideal health professionals to deliver such an intervention as they have regular contact with women throughout pregnancy providing multiple opportunities for them to intervene. Studies have reported that pregnant women feel health professionals should support and guide them about weight gain during pregnancy and that midwives were the most appropriate people to do so [24]. Studies show women believe that if gestational weight gain was important for a healthy pregnancy their midwife would have raised the issue [19], which presumably leads pregnant women to conclude it is not a health risk to them or their baby if their weight is not discussed or monitored by health professionals. One intervention that has shown promise in helping people manage their weight is regular weighing, a form of self monitoring [25, 26]. Some countries routinely weigh pregnant women as part of antenatal care (e.g. USA, Canada) but many do not (e.g. UK, Australia, New Zealand, Ireland) [27]. Even in countries that do weigh women routinely, none have a policy of setting regular weight gain limits and encouraging women to weigh themselves weekly to assess their gestational weight gain progress. In the UK there has been growing interest in the possibility of routine weighing of women in pregnancy but the National Institute for Health and Care Excellence (NICE) [28, 29] do not currently recommend that pregnant women are weighed regularly or given information on optimal gestational weight gain as there is insufficient evidence of effectiveness. There is also no evidence that regular weighing of pregnant women does not cause harm either (e.g. maternal anxiety).

The primary aim of this trial is to examine the feasibility and acceptability of an intervention in which community midwives routinely weigh women throughout pregnancy, set the parameters for healthy gestational weight gain, provide progress feedback at each antenatal appointments and encourage women to self monitor their own weight gain by weighing themselves weekly between antenatal appointments.

## Methods

### Intervention development

Our hypothesis is that engagement in regular weighing and setting maximum weight gain limits and feedback on gestational weight gain progress may improve dietary vigilance; documenting weight gain against the parameters set for this may raise individuals’ awareness of the behaviours that influence their weight and encourage them to take action if it is required. This hypothesis is driven by the principles of self regulation theory and the relapse prevention model [30, 31]. An individual who is regularly weighed (either by themselves or someone else) is more likely to stay focussed on changes in their weight and this provides opportunities to identify lapses in their progress, reinforcement of progress and stimulate behavioural adjustments to achieve weight goals before these are unattainably out of reach. Although NICE in the UK [28, 29] do not currently recommend that community midwives, or any other health professional, routinely give information about optimal weight gain during pregnancy, data in non pregnant populations suggest that frequent monitoring of body weight is associated with improved weight control [32–34]. The focus of this intervention is to help pregnant women acknowledge their weight gain thus far, and to consider strategies to respond appropriately if excessive weight gain occurs.

### Design and design considerations

This study is a two group (individual) randomised controlled trial. Recruitment took place between April 2012 and December 2012 with all participants completing follow up by September 2013. Ethical approval for the study was issued by South Birmingham National Research Ethics Service (ID: 12/WM/0059) in March 2012. Written informed consent was obtained from all participants prior to randomisation.

We considered whether this feasibility trial should be a cluster randomised trial. The basis of the intervention is that a weight gain chart is added to the maternity notes. This does not occur in the usual care group so there is no reason or trigger for midwives to weigh women and no chart in the notes on which to offer feedback or have a conversation, except for those in the intervention group. This led us to conclude that there may not be an issue with contamination in this trial. In short, there appears little motivation for midwives to offer usual care women the intervention proposed. A key concern that arises in many cluster trials is that they lead to potentially biased recruitment. In this trial community midwives will identify potentially eligible women. If we used a cluster trial design it would mean midwives would know from the outset which cluster they had been allocated to and would know in advance the group allocation of women they identified as eligible. This might mean that midwives in the control cluster are less keen to recruit women because they know their women will not receive the intervention, leading to differential recruitment to the groups, undermining the integrity of the trial. This very problem has occurred before in cluster trials where midwives were responsible for recruitment [35]. In addition, midwives in the intervention group might only recruit women who they expected to adhere to the intervention. Given that we suspected that there may be little contamination, we designed this feasibility trial to specifically examine whether contamination occurred. If it does, we would then need to consider its impact when planning a phase III definitive trial should the intervention prove feasible and acceptable here.

### Study participants and recruitment

Low risk pregnant women receiving community midwife led care, aged ≥18 years and within healthy or overweight BMI ranges (18–29.9 kg/m^2^) at their first antenatal appointment (6–8 weeks of pregnancy were potentially eligible. Obese women (≥30 kg/m^2^) were not eligible as the focus of this study was primary prevention and because many obese pregnant women already receive additional weight management support which we did not want to interfere with our intervention. In the maternity centre used in this study women with a BMI of ≥40 kg/m^2^ are considered at high risk of complications during pregnancy and routinely receive consultant/physician led care and not community midwife led care. Other women deemed at high risk of complications (therefore receive consultant/physician care) by the community midwife at the first antenatal appointment were also ineligible and not invited to take part. Participants were recruited from one maternity centre in England.

Community midwives introduced the study to women thought to be having a low risk pregnancy and potentially suitable for the trial at their first antenatal appointment (6–10 weeks of pregnancy). At this time, community midwives handed women the study information leaflet, asked them to read it and advised them that they might be invited to participate in a study about weight gain during pregnancy immediately after their dating/booking scan at 10–14 weeks of pregnancy. Women confirmed as having a low risk pregnancy at their 10–14 week booking scan were then approached, recruited and randomised by the research team.

### Intervention

No official clinical guidelines for weight gain during pregnancy exist in the UK [28, 29]. We developed an intervention to prevent excess weight gain in women at low risk of obstetric complications but which community midwives would offer all women. Midwives biggest concerns about addressing prevention is about the time it might take. With these considerations, we designed an intervention that could be delivered in less than two min yet could still be effective. In addition, it has to be acceptable to women. The intervention supplemented usual maternal care.

**Fig. 1:**
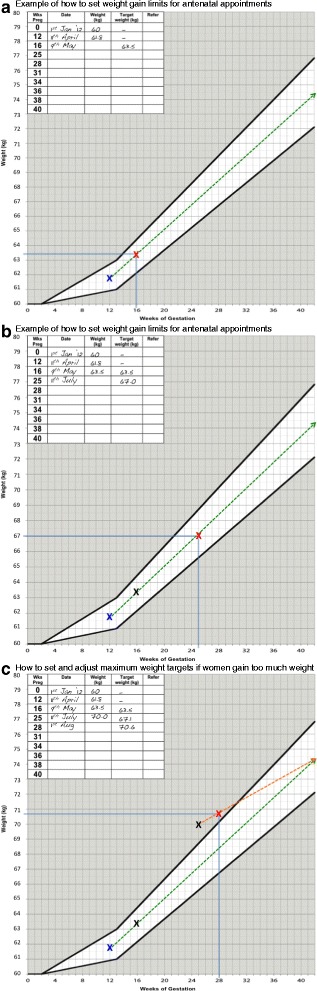
**a** The woman is recruited at 12 weeks gestation and her weight is plotted on the chart for this week of pregnancy. The woman is advised that her weight should follow the dotted line drawn through the ideal weight gain zone on the chart (unshaded area). The woman is due to be seen again by her midwife at 16 weeks gestation therefore the midwife draws a vertical line at 16 weeks gestation to meet the dashed line in the unshaded ideal weight gain zone to ascertain what the maximum weight target should be for 16 weeks gestation. In this example the woman is advised by her midwife that ideally her weight should be no more than 63.5 kg at 16 weeks gestation. The midwife repeats the procedure at each antenatal appointment. **b** At 16 weeks of pregnancy the midwife weighed the woman and plotted her weight on the chart. In this example the woman weighed 63.5 is 16 weeks gestation which was the maximum weight limit set at her previous appointment at 12 weeks gestation. The midwife then set the maximum weight target for the next antenatal appointments which was scheduled for 25 weeks gestation. The midwife draws a vertical line at 25 weeks of gestation to meet the dashed line in the unshaded ideal weight gain zone to ascertain what the maximum weight target should be for 25 weeks gestation. In this example the woman should ideally weigh no more than 67 kg at 16 weeks gestation. **c** At 25 weeks gestation the midwife weighed the woman and plotted her weight, which was 70 kg. This was above the maximum weight target set at the previous appointment at 16 weeks gestation. The midwife therefore redraws the ideal weight trajectory line starting from the plotted weight at 25 weeks gestation to the central point in the unshaded weight zone until 42 weeks gestation. The midwife uses this new line to set the maximum weight target for the next antenatal appointment scheduled for 28 weeks gestation. The midwife draws a vertical line at 28 weeks of gestation to meet the dashed line in the unshaded ideal weight gain zone to ascertain what the maximum weight target should be for 28 weeks gestation. The midwife advised the woman that her maximum weight target for 28 weeks of pregnancy was 70.6 kg.

The intervention involved several interrelated components. Community midwives were asked to weigh women at each antenatal appointment (up to eight times) and plot their weight on an Institute of Medicine (IOM) [4] weight gain chart (see Fig. 1), specific to their pre-pregnancy BMI category. The chart was attached to the hand held pregnancy notes and outlined a maximum weight gain limit for their next appointment, for the women and midwife to assess weight gain progress. Women were given feedback from their community midwife on their progress emphasising the importance of weight gain but within a limited healthy range. The explicit behavioural goal of the intervention was for women’s weight gain to follow the trajectory of the midpoint line in the threshold zone of their IOM chart (see Fig. 1). Consistent with clinical advice the goal was always for weight gain and never weight loss. Women were also given a weight record chart and asked to weigh themselves weekly and record these weights to monitor their own weight gain progress.

In the UK clinical guidance [29] stipulates for a woman who is nulliparous with an uncomplicated pregnancy, a schedule of 10 appointments should be adequate. For a woman who is parous with an uncomplicated pregnancy, a schedule of seven appointments should be adequate. As recruitment was planned to take place around 10–12 weeks of pregnancy when two appointments will have already taken place, we expected women to receive the intervention from their midwife up to eight times during pregnancy.

We set parameters for healthy weight gain for each subsequent antenatal appointment and encouraged women to stay within the two threshold lines on the weight chart (ideally following the midpoint line through the threshold). Community midwives were taught how to adjust women’s maximum weight gain limit if they were off track, but safely and slowly; see Fig. 1 for a visual explanation of this process. Women whose weight gain was within the appropriate range on the chart were told they were gaining the ideal amount of weight and encouraged to maintain a healthy lifestyle. Women gaining too much weight were encouraged to eat a healthy diet and restrict their intake of high fat and sugary foods and drinks and to participate in regular physical activity (walking). Women gaining too little weight, and those who exceeded their maximum weight gain limit as set by their community midwife on three consecutive occasions, were referred to the appropriate health professional for additional support in line with local practice.

As this was intended as a brief intervention that could be implemented into routine antenatal care, community midwives were not expected to engage women in detailed lifestyle counselling about how changes to diet and physical activity might be implemented; the focus was on giving brief feedback and advice in line with current NICE guidance [28]. As part of the feedback from the weight gain charts, community midwives gave messages around the importance of preventing excessive weight gain during pregnancy and addressed myths and misconceptions about eating and exercise behaviours during pregnancy (e.g. “eating for two”, “weight gain does not matter when you are pregnant”, “you shouldn’t exercise when you are pregnant”). Women were encouraged to accumulate 30 min of moderate intensity physical activity (walking) each day in line with current recommendations [36].

### Usual care

The usual care group received standard maternity care according to local health care provision and no other intervention. This is not a trial about giving lifestyle advice therefore community midwives were not asked to refrain from offering usual advice about diet and exercise early in pregnancy.

### Training of community midwives to deliver the intervention

Conscious that only interventions requiring a short training course would ever be widely implemented in routine antenatal care, we designed a 60–70 min course for delivery to a group of community midwives. The training manual contained information on study eligibility criteria, recruitment procedures and the importance of adhering to protocol and study design and not contaminating usual care. Information on the consequences of weight gain during pregnancy, instructions about how to weigh and plot weight on the IOM weight chart and how to give feedback on the weight gain chart and example messages were also outlined. Explanation of how to set weight gain limits using the charts and examples of educational and motivational messages that should be given about gestational weight gain, diet and physical activity during pregnancy were also included. Midwives also practiced completing the weight gain charts using prepared case studies.

### Outcomes

The primary outcome of this trial was the feasibility and acceptability of the intervention to women with a focus on process and formative evaluation in line with the MRC Framework for developing complex interventions [37]. Accordingly the proportion of women that were referred per midwife/month, randomised, refused and drop-out rate were recorded and the experience of the trial from the perspective of community midwives and participants was assessed. This feasibility study was not designed to detect differences in weight gain. However, it would be remiss not to record weight change and the proportion of women in each group who exceeded the IOM guidance was designated as a secondary outcome and to inform a power calculation for the definitive trial. Other outcomes included physical activity [38] depression and anxiety [39]. Data regarding the occurrence of serious adverse events requiring hospitalisation were collected.

### Intervention fidelity and process outcomes

Two weight gain charts from each midwife were checked at the beginning of the intervention period for accuracy and completeness by the research team when women were about 20 weeks pregnant. If inaccuracies were noted the research team contacted the community midwife to discuss these to ensure they did not occur in the future. The weight gain charts were retrieved from the hand held pregnancy notes after women had delivered their baby and were assessed for accuracy and to provide a measure of intervention implementation. From the weight gain chart we checked whether weight had been measured, plotted and recorded by community midwives and if maximum weight gain limits were calculated and recorded correctly at each antenatal appointment.

### Assessment of outcomes at baseline and follow up

Weight was assessed using calibrated scales. Height and weight were measured with excess clothing and shoes removed. Weight, height, and hence BMI is routinely assessed as part of standard maternal care at 6–10 weeks of pregnancy and recorded in the hand held pregnancy notes (i.e. pre baseline measure in this study) in the participating hospital. Weight was measured again at 12–14 weeks (recruitment/baseline), 38 weeks of pregnancy, as well as 72 h and 6–8 weeks postnatally in all participants either by the research team or community midwives. These data allowed us to calculate total gestational weight gain and weekly average weight gain. The number of participants per group who remained within the IOM guidelines for their pre pregnancy BMI category was calculated.

The study questionnaires were mailed to participants at baseline and at each follow up and collected at home visits by the research team or returned by post. The questionnaire also assessed the advice given to women throughout pregnancy by their community midwife about weight control, eating, and physical activity; it assessed whether community midwives were giving the intervention group the type of messages that were in line with the training provided. In the usual care group the questionnaires assessed intervention contamination. Demographic and weight-related data were collected on age, marital status, ethnicity, social class, parity, smoking status, employment status was also collected. After two weeks of no response one reminder was sent to women who did not complete and return their study questionnaire pack.

### Criteria for determining the need for a subsequent phase III definitive trial

The criteria for concluding the study and intervention were feasible and acceptable were that at least 50 % of gestational weights and maximum targets for weight gain were recorded on the weight charts in line with the training provided and at least 30 % of eligible women were recruited and findings from the interviews (see below) did not oppose progression to a phase III trial. In health behaviour research it can often be difficult to give a precise definition or cut-off for when behaviour is deemed acceptable or not and a reasonable judgement on what such a cut-off might be in any given context or population need to be made. We selected the criteria that at least 50 % of gestational weights and maximum targets for weight gain needed to be recorded on the charts by midwives to deem the intervention feasible because it meant at least half had been implemented correctly. We wish to make it clear here that this 50 % criteria was chosen as the minimum criteria. We selected a 30 % uptake rate in eligible women as the minimum criteria for determining acceptability in women because this would mean about one in every three eligible women approached were randomised; this rate seemed reasonable as a minimum criteria on which to determine acceptability.

### Blinding, randomisation and allocation concealment

The randomisation list was generated by the trial statistician (AR), independent from researchers involved in recruiting and randomising participants. Participants were randomised on a 1:1 basis to intervention or usual care using random permuted blocks of mixed size (2, 4 or 6) within strata (midwife). The researcher allocated women by opening sequentially numbered opaque sealed envelopes. The researcher opened the envelope after eligibility assessment. Because of the nature of the intervention, participants, researchers and those delivering the intervention could not be blinded to group allocation.

### Sample size

As this is a feasibility study formal power calculations were not appropriate. The sample size was determined by the need to gain sufficient understanding of the recruitment processes and an adequate range of participants’ experiences of the intervention. We initially aimed to recruit 90 pregnant women referred from eight community midwives in diverse areas and randomised to receive usual care only or usual care plus the intervention. We expected 20 % to drop-out resulting in data collected on 72 women at 38 weeks of pregnancy.

### Qualitative study: Feedback from women and community midwives

#### Participants

At the 6–8 week postnatal home visit the intervention group were invited to complete a semi structured interview about their experiences of participating in the study. Purposive sampling was used to ensure a range of women were included (age, ethnicity, socio-economic status, number of children, healthy weight/overweight at randomisation). The topic guide explored women’s general experiences of study participation, their understanding of the importance of weight management in pregnancy, how they tried to manage their weight, as well as what they thought about being weighed by their community midwife. Women were specifically asked about the emotional impact of being weighed regularly.

#### Community midwives

Once all the women in their care had delivered midwives completed a semi structured interview about their experiences of delivering the intervention and views on the study as a whole. The topic guide explored midwives experiences of study participation, reflections on the potential role of regular weighing and whether it is feasible to deliver during antenatal care for pregnant women, the aspect of the intervention they found easy/difficult to deliver, how they felt about raising the topic of weight gain, whether they felt the intervention made women anxious, how the intervention affected the dynamics and length of consultations and we asked for feedback on how the intervention could be improved.

#### Data analysis

As this is a feasibility trial that was primarily concerned with developing an intervention we have presented descriptive information in the form of percentages, means and standard deviations for the quantitative outcomes of interest for the trial groups. Accuracy of completion of the weight gain charts by community midwives in line with the training provided was assessed using three criteria; (1) was weight plotted and recorded correctly on the chart at each appointment?, (2) were the maximum weight gain targets recorded on the weight chart at every appointment?, and (3) were the maximum weight targets set accurately at each appointment? Each chart was scored according to these criteria and allocated a percentage intervention accuracy score.

The interviews were recorded and transcribed with the permission of participants and thematically analysed using a constant comparative method. A thematic approach was taken to categorising the data to identify emerging issues within each theme. These were compared, discussed and organised by the same researchers (AD & SC). The existence of thematic categories was validated by two raters (AD & SC) being reliably able to allocate responses to these particular category headings.

## Results

### Trial flow and recruitment

**Fig. 2:**
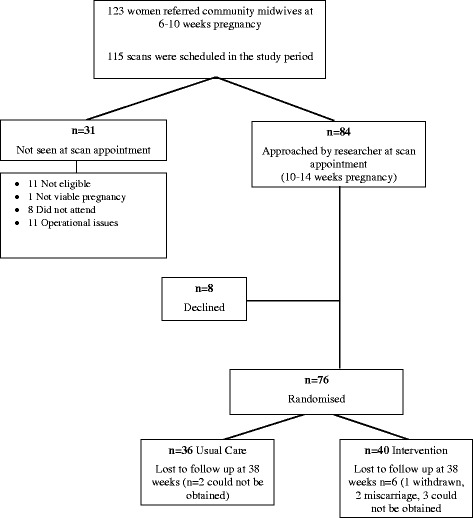
Trial flow

Eight community midwives referred 123 women to the study team who they believed to be having a low risk pregnancy at the 6–8 week antenatal appointment and 115 were scheduled for their dating scan (10–14 weeks of pregnancy) within the study period. It was not logistically possible for us to approach all 115 because some women were due to have their dating scan to confirm their eligibility during the University Christmas holidays/closed days, but of those approached at their scan (*n* = 84) 76 women (76/84, 90.4 %) agreed to participate and were randomised. Reasons for non recruitment are stated in Fig. 2.

Randomised participants were on average 28.5 years, most lived in areas in the highest two quartiles of multiple deprivation index (63.2 %), 11.8 % were of non white ethnicity, 56.6 % were healthy weight and 43.4 % overweight and 46.1 % had no previous children. These characteristics were generally balanced across the trial groups except for ethnicity and null parity where there was imbalance (Table 1). Over 90 % of women completed follow up of weight at 38 weeks of pregnancy and 72 h after birth and 89 % completed follow up of weight at 6–8 weeks after giving birth. Two participants were referred to another health professional because they had gained excessive gestational weight gain on three consecutive occasions in line with the study protocol but they continued with the study. Questionnaire completion rates across follow up ranged from 62–74 %. Eighty five percent of the weight charts retrieved (*n* = 35/37) had been completed with at least 80 % accuracy, meaning that weight was plotted accurately and targets set as we had instructed. Most women had a normal vaginal delivery (intervention: 35/36, 97.2 %; usual care: 32/37, 86.5 %).

**Table 1:** Characteristics of randomised participants (*n* = 76)

	Usual care		Intervention	
Mean Age (SD)	28.9	(6.8)	28.1	(5.9)
IMD (%)				
Quartile 1(least deprived)	7	(19.4)	8	(20.0)
Quartile 2	8	(22.2)	5	(12.5)
Quartile 3	8	(22.2)	15	(37.5)
Quartile 4 (most deprived)	13	(36.1)	12	(30.0)
Smoked (%)	12	(33.3)	9	(22.5)
Not known	2	(5.6)	3	(7.5)
White ethnicity (%)	33	(91.7)	34	(85.0)
BMI category (%)				
Healthy weight (BMI 18.5–24.9)	20	(55.6)	23	(57.5)
Overweight (BMI 25.0–29.9)	16	(44.4)	17	(42.5)
Previous children (%)				
None	10	(27.8)	25	(62.5)
One or More	26	(72.2)	15	(37.5)
Marital status (%)				
Married	15	(41.7)	21	(52.5)
Single (living alone)	2	(5.6)	2	(5.0)
Single (living with partner/family)	17	(47.2)	17	(42.5)
Divorced (living with partner)	1	(2.8)	0	(0.0)
Not known	1	(2.8)	0	(0.0)
Employment status (%)				
Employed	29	(80.6)	35	(87.5)
Unemployed	5	(13.9)	3	(7.5)
Student	1	(2.8)	1	(2.5)
Looking after family	0	(0.0)	1	(2.5)
Not known	1	(2.8)	0	(0.0)

### Excessive gestational weight gain

Table 2 shows a small difference favouring the intervention group in the percentage of women exceeding the IOM recommended weight gain at 38 weeks of pregnancy (23.5 % versus 29.4 %). This was more pronounced for overweight women (37.5 % versus 53.5 % respectively) than for women with a healthy pre-pregnancy BMI. Total weight gain at 38 weeks of pregnancy and average weekly weight gain were similar in both groups.

**Table 2:** Weight outcomes at follow up

	Usual care		Intervention	
Randomised	36		40	
Withdrawn	0		1	
Miscarriage	0		2	
38 week weight not obtained	2		6	
Weight gain (pre-booking to 38 weeks gestation *n* = 68)				
% exceeding recommended weight gain	10/34	(29.4)	8/34	(23.5)
Healthy BMI category	2/19	(10.5)	2/18	(11.1)
Overweight BMI category	8/15	(53.3)	6/16	(37.5)
	Mean	(sd)	Mean	(sd)
Average total weight gain (kg)	12.1	(5.9)	12.0	(4.5)
Healthy BMI category	12.6	(5.1)	12.3	(4.0)
Overweight BMI category	11.6	(7.0)	11.6	(5.1)
Average weekly weight gain (kg)^a^	0.4	(0.2)	0.4	(0.2)
Healthy BMI category	0.4	(0.2)	0.4	(0.1)
Overweight BMI category	0.3	(0.2)	0.4	(0.2)
Weight gain (pre-booking to 72 hours post delivery *n* = 69)				
Average Total Weight Gain (kg)	6.6	(5.8)	7.5	(4.7)
Healthy BMI category	7.1	(5.4)	7.4	(4.4)
Overweight BMI category	6.0	(6.3)	7.5	(5.2)
Weight gain (pre-booking to 6–8 weeks post delivery *n* = 65)				
Average total weight gain (kg)	3.1	(5.4)	4.4	(4.5)
Healthy BMI category	4.0	(4.9)	4.4	(4.0)
Overweight BMI category	2.0	(5.9)	4.4	(5.1)

^a^IOM guidance: women in the healthy and overweight BMI categories at the start of pregnancy should gain between 0.35–0.5 and 0.23–0.33 per week of pregnancy

### Physical activity, depression and anxiety and serious adverse events

The intervention group reported more total physical activity at 38 weeks of pregnancy and 6–8 weeks postnatally but less at 28 week of pregnancy than usual care (Table 3). The intervention group also reported substantially more (double the rate) vigorous intensity physical activity than usual care at 28 and 38 weeks of pregnancy. The intervention group consistently reported smaller increases in depression and anxiety scores throughout pregnancy compared with usual care (Table 4). There were no serious adverse events requiring hospitalisation.

**Table 3:** Pregnancy physical activity questionnaire (MET minutes per day)

	Usual care			Intervention		
	Number	Mean	(sd)	Number	Mean	(sd)
Total Activity:						
12 weeks	34	2665.9	(1214.4)	38	2634.6	(1211.3)
28 weeks	21	2242.3	(1128.1)	31	2136.1	(1247.6)
38 weeks	22	1831.7	(843.6)	17	1964.7	(1356.2)
Postnatal	27	2337.2	(725.3)	24	2417.9	(760.1)
Vigorous:						
12 weeks	35	23.7	(44.4)	39	29.3	(54.8)
28 weeks	23	12.8	(29.7)	31	25.1	(49.1)
38 weeks	24	8.2	(16.0)	18	20.1	(28.8)
Postnatal	27	34.5	(55.0)	25	24.2	(30.8)
Moderate:						
12 weeks	35	1098.6	(1003.4)	38	972.2	(770.3)
28 weeks	22	784.7	(728.4)	31	623.6	(821.2)
38 weeks	23	512.7	(488.0)	18	534.6	(749.8)
Postnatal	27	927.7	(472.4)	25	1039.9	(731.3)
Light:						
12 weeks	34	1056.8	(511.2)	38	949.4	(609.6)
28 weeks	22	948.8	(532.3)	31	813.1	(514.1)
38 weeks	23	830.9	(475.0)	17	777.4	(504.5)
Postnatal	27	1055.2	(340.3)	24	1131.8	(355.9)
Sedentary:						
12 weeks	35	564.2	(228.8)	39	683.4	(312.9)
28 weeks	22	528.5	(231.0)	31	674.3	(335.1)
38 weeks	23	451.6	(197.0)	18	616.8	(393.9)
Postnatal	27	319.8	(179.2)	25	325.4	(202.9)

Total physical activity scores are based on all subscales of the PPAQ including household, work and sport but the individual scores for these subscales not reported. 12 weeks refers to baseline assessment

**Table 4:** Hospital anxiety and depression scale

	Usual care			Intervention		
	n	Mean	(sd)	n	Mean	(sd)
Anxiety:						
12 weeks	34	5.4	3.4	39	4.8	2.8
28 weeks	23	6.4	3.5	31	4.7	2.7
38 weeks	24	5.7	3.0	20	4.4	2.7
Postnatal	24	5.7	3.0	20	4.4	2.7
Depression:						
12 weeks	34	2.8	2.4	40	3.1	2.5
28 weeks	24	4.8	3.3	31	3.4	2.6
38 weeks	24	5.4	3.4	20	3.8	2.7
Postnatal	24	5.4	3.4	20	3.8	2.7
HADS score:						
12 weeks	34	8.2	5.0	39	7.9	5.0
28 weeks	23	11.0	6.2	31	8.2	4.4
38 weeks	24	11.1	5.7	19	8.5	4.6
Postnatal	27	7.7	4.9	24	7.6	3.9

12 weeks refers to baseline assessment

### Questionnaire items about the healthy living messages given to women by midwives

Questionnaires completed by participants at the end of each trimester indicated that midwives were giving women in the intervention group healthy living messages in line with the training we had provided. In the usual care group we found no evidence of contamination; midwives were not routinely weighing women, were not charting weight as there is no weight chart in the notes. There was no evidence midwives were offering any enhanced advice about healthy lifestyles to the usual care group. Typically usual care reported that their midwife had told them to eat sensibly and had given them advice about iron rich foods and avoiding listeria. Examples of the kinds of advice usual care participants reported receiving from their midwife were “not weighed by midwife but she commented that I was 'all bump”, “after I asked her she gave me guidance on safe exercise”, and “avoiding foods I shouldn't eat while pregnant”.

### Qualitative outcomes

Thirteen women were invited to participate in the study and 12 agreed (Table 5). Results are presented under key themes/questions from the interview schedule. Quotations reflecting the range of issues that emerged are presented and were selected because they were typical of the insights that participants gave during interviews. The number of respondents who had mentioned specific issues within each theme is included to help illustrate which issues arose most frequently among participants [40].

**Table 5:** Interviewee characteristics

Midwives *n* = 7	
Experience	Range = 6–29 years (mean 18.3)
Full time	6
Participants *n* = 12	
Age	Range = 19–41 years (mean 29.3)
Ethnicity	
• White	9
• Pakistani	2
• Thai	1
Parity	
• First Child	9
• Second Child	2
• Third Child	1
BMI	Range = 19.0–29.8 (mean 24.3)
IMD Quartile	
• 1	1
• 2	3
• 3	5
• 4	3

### Interviews with women

#### Thoughts about the intervention

The majority (*n* = 9) commented they found the intervention useful because it helped them to monitor their weight gain and to be more vigilant about what they were eating and how much physical activity they were completing each week. Two did not find the intervention useful and did not refer to the weight gain chart regularly throughout pregnancy and one was unsure. Nine participants emphasised that the value of the intervention was in terms of providing a source of motivation to think more closely about their weight than might have been the case otherwise. One participant commented that the intervention was not at all motivating because she found her weight was under her target weight set by the midwife.

*It made me think more so about what I was eating, as opposed to just eating everything I could see, but I was very conscious to eat, eat properly as well*

*I didn’t really look at them to be honest. I mean it was interesting to see how much I was gaining obviously, because then you can look back and can think, you know, in 9 months I put on 20 pounds or whatever, but*

*R: So on a weekly basis you didn’t look at the chart?*

*I didn’t look at the chart, no*

*R: So you didn’t find it motivating because you were under weight rather than over.*

*Yeah sometimes I found it a bit, not depressing but, I found it a bit harder.*

#### Being weighed and talking to the midwife about weight gain

All of the participants interviewed felt comfortable talking to their midwife about their weight. Of interest here, one participant commented that she may have found these conversations more difficult had she been gaining weight excessively. Ten participants responded very positively to the importance of being weighed regularly during pregnancy, one participant was positive with some caution and one responded positively but felt women should not be weighed at every appointment. Most participants (*n* = 7/12) reported they had weighed themselves between appointments but of these only three recorded their weight on the record card given to them.

*Yes, definitely. I was really surprised that they didn’t and I definitely think it would be useful. A lot of my friends, when I said that I was on it, who are also pregnant said oh my god that’s really good, I wish my midwife did that.*

#### Feelings about being weighed

Eight participants indicated they had not been anxious at all, three felt a small amount of anxiety and one participant reported she felt anxious towards the end of pregnancy when the midwife informed her that she had exceeded her target. Six participants were clear that the intervention had not led them to be unduly anxious and two commented it had but gave no further explanation. One participant commented the intervention had probably made her more anxious because normally she would not have had information about her weight gain progress, one participant said she would have worried regardless.

*I think I was more aware of it, I’m not sure it made me anxious, probably just more aware.*

#### Describing your experiences to other pregnant women

Nine participants commented that if they were asked to describe their experiences of study they would emphasise the intervention was a positive experience for them and that the intervention was a good idea because it helped them to monitor their weight gain and because they received additional advice. One participant would advise women to ignore the targets and carry on with their lives in the normal way (P7) and another suggested that they had taken the intervention *“with a pinch of salt”* (P6).

*Just that it’s a good check and good advice really for what to eat and what not to eat and I think some people do think you’re pregnant, you eat for two and don’t realise the aftermath and what weight you’ve got to take back off.*

*I did find it helpful in the fact that it kept my mind focussed on where my weight was but the other side of me I did take it a bit with a pinch of salt. I still ate but I think if anything it just gave me more motivation to go out and go walking and be more active, but then I wasn’t trying to lose weight.*

#### Interviews with community midwives

Seven of the eight community midwives were interviewed. Midwives ranged in years of experience (6 to 29 years) (Table 5).

#### Discussing gestational weight gain

None of the community midwives expressed any difficulties broaching the issue of gestational weight gain with women and reported that women themselves did not mind being approached to participate and were very positive about being involved in the study. Midwives felt that they frequently had to have ‘difficult’ or sensitive conversations and this was part of their every day job and raising gestational weight gain was seen in these terms. Midwives expressed mixed views on the importance of discussing gestational weight gain with women; six said they considered it to have medium to low priority but that they would give it higher priority if women were overweight or obese.

#### Regular weighing

All the midwives reported there were no problems with weighing the intervention group at their routine appointments and women were ‘*eager to jump on the scales’* and ‘*expected to be weighed’*. There was acknowledgement from midwives that weight was a sensitive topic but nonetheless that the intervention was easy to deliver. Six out seven midwives said they thought routine weighing was important. One was unsure because she had some concern about women who might not be gaining sufficient weight and this might lead to them having a small baby but this midwife still felt midwives should weigh women during pregnancy to help reduce obesity.

*It’s having the, I suppose the confidence to broach it with them in the first place. Because you have loads of talking and training whatever about smoking and this, that and the other, but when it comes to weight management you don’t get much training about it really.*

#### Women’s feelings about regular weighing

Only one midwife reported that one of her women was a little anxious about her weight. One midwife commented that as a midwife she should be able manage anxiety about any aspects of pregnancy that women might have. Two midwives suggested that anxiety might be more common in obese women rather than overweight and healthy weight women.

*A lot of them were really quite positive about it, like “Yes please, I want to be weighed every week” and I was going like that’s not possible, it might be an option, but it might not be an option. So no, mine were really all quite positive about it.*

*R:And do you remember any negative responses*

*No. Not off any of mine*

*Yes, yes and in fact the girls that were, you know on the study, they were expecting it and they did know what their target weight was for the next time, so they were more eager to jump on the scales in the end...... So in that point it was quite good because they were more wary of it weren’t they really, the end of the pregnancy it made them more aware, so it wasn’t a problem*

*I think that you may get that [anxiety] with someone with someone who was obese, with a BMI above 30, you know what I mean*

*R:They’re not in the study*

*They’re not in the study so we never had to address that in such detail, you know what I mean*

*No I don’t think so* [referring to anxiety in women] *because I only had one that fell sort of out of her normal range, there might have been two, and she was so blasé about it that she wasn’t remotely stressed at all....There are plenty of things for ladies to be anxious about in pregnancy and it is your job to try and stop them from being you know anxious, you should be able to recognise that I think and try and put them at their ease the best you can....*

#### Delivering the intervention in routine care

Midwives did not think the intervention added substantially to their consultation length, with the majority indicating the intervention took one-three min at the most and that intervention could be incorporated into a standard 10–15 min appointment slot. The additional time for the intervention was not perceived as problematic by midwives. Five out of seven midwives commented that they did believe it was possible for a community midwives to routinely deliver the intervention to all pregnant women.

*Oh it was probably a matter of a minute, you know, just kind of stick them on the scales and, well maybe three minutes if you include like the drawing their lines on and everything. Yeah it really wasn’t long at all.*

*So sometimes it’s just as you say, it’s having the, I suppose the confidence to broach it with them in the first place. Because you have loads of talking and training whatever about smoking and this, that and the other, but when it comes to weight management you don’t get much training about it really, as such.*

#### Overall thoughts about the intervention

Midwives highlighted a number of things that they would convey to their colleagues about the study. Midwives felt the study provided an opportunity to talk about weight/diet/physical activity with women, the intervention should be provided to “*bigger ladies”,* that prevention was better than cure and therefore the study was a good idea. Some midwives commented that the role of the midwife has become more public health orientated and managing gestational weight fitted with this role. Midwives also commented that they would have liked a specialist senior community midwife to be more highly trained in weight during pregnancy who they could contact if they had any questions or concerns.

*It prompts you, it reminds you for a start to weigh them, that kind of reminds you to talk about their diet… you know you talk to them about how they’ve been, you know, so that whole public health agenda… and giving you the opportunity as well to have the conversation.*

*I think it makes people more aware of their weight during pregnancy. I think it makes them, because they know that they’re going to be weighed you know in the consultation I think it does make them more aware because, people you know when they were being asked to be weighed they’ll go ‘oh I have been really good this week, haven’t had any chocolates’ or you know, so I do think it had a positive effect to be honest. So it’s something else for us to do but I think it did have a positive impact on the ladies and that’s what we’re all here for isn’t it at the end of the day.*

## Discussion

Adding regular weighing, weight gain limits, feedback and encouragement to weigh weekly is a feasible and acceptable addition to routine antenatal care provided by community midwives. Of those fully eligible and approached at their booking scan, 91 % (*n* = 76/84) agreed to participate and were randomised indicating that women were keen to participate in a study that they were aware would involve being weighed and given feedback by their midwife. Our recruitment rate is in line with studies conducted in other countries that have incorporated lifestyle interventions into the routine antenatal care of pregnant women [41]. Studies that have not embedded their interventions within routine antenatal care have reported much lower recruitment rates. For example, the LIMIT [42] trial, which randomised overweight/obese pregnant women to a dietary and lifestyle intervention or usual care, reported that only 40 % of those eligible agreed to participate. Our fidelity checks showed that midwives were able to deliver the intervention with a high level of accuracy, demonstrating the training module we developed worked well and that interventions of this kind have potential for very high coverage and implementation. Data collected throughout the intervention showed no evidence of intervention contamination in usual care.

This trial was not large enough, nor intended, to estimate the difference in proportion of women who exceeded the IOM guidance which awaits an effectiveness trial. There was evidence that the intervention group had a slightly higher (6 %) proportion of women achieving healthy weight gain. Using the UK as an example, if offered to all pregnant women this would mean the intervention which is brief, could prevent 49,000 women per year from gaining excessive weight gain during pregnancy, assuming the current birth rate of 813,000 [43]. There was no evidence the intervention caused anxiety in the intervention group relative to usual care. Physical activity scores tended to favour the intervention group compared with usual care showing the intervention group had in acted the advice given to them by their midwife.

### Views of pregnant women

Women allocated to the intervention group reported their main reason they liked the intervention was that it might help them avoid gaining too much weight during pregnancy and it would be useful in helping them to be more vigilant about their eating and physical activity. This result differs from other qualitative studies that have reported women did not worry about gestational weight believing they would lose it postnatally [19, 24]. This could be because midwives repeatedly stressed that women in the intervention group should not gain too much weight which was not the case in the other studies. This suggests that such repeated simple messages may change women’s beliefs.

Women particularly liked being weighed at each appointment and receiving specific feedback because it provided ongoing motivation to consider their lifestyle choices. Another qualitative study has also reported that pregnant women acknowledged that if they were weighed regularly it would be easier to ensure they did not gain too much weight [19]. Many studies [19, 24, 44] have reported that pregnant women feel they receive inadequate advice from healthcare professionals regarding diet and physical activity; adding regular weighing within routine antenatal care may be a vehicle for ensuring pregnant women receive this information universally.

Most of the women interviewed (58 %) weighed themselves each week between antenatal appointments, but the majority did not record this in their record chart (25 %). Recording information is an important part of self regulation [30] since it provides the opportunity for reflection on progress and to take action if required. Thus, it appears the intervention was not entirely successful in getting pregnant women to engage in all components of self monitoring and there was a reliance on midwives for feedback on progress. There can be several weeks between antenatal appointments and if women do not weigh themselves and wait until they are next seen by their midwife to be weighed it may be too late for them to take action and excessive gestational weight may have occurred. If women work more in partnership with their midwife to monitor their weight gain this would probably improve the effectiveness of the intervention and help women to develop self-management strategies. Any future trial will need to incorporate enhanced strategies that more actively involve pregnant women in self monitoring and managing their own gestational weight.

### Views of community midwives

Midwives felt comfortable and confident about raising the issue of gestational weight gain. This is encouraging since other studies have reported that midwives can be reluctant to discuss weight gain [45]. Community midwives commented that they would give it higher priority in overweight/obese women. Midwives felt routine weighing of pregnant women by community midwives was important to do to make women more aware of their weight gain during pregnancy. This is consistent with previous studies that have suggested that health professionals feel gestational weight gain should be monitored [45, 46]. Midwives felt women responded well to the intervention and it did not feel it made women unduly anxious; this is in line with the quantitative data we collected which showed the intervention group reported lower anxiety and depression scores throughout pregnancy than usual care.

All the midwives felt it was feasible to deliver the intervention within the context of routine antenatal care, taking about one-three min per appointment to deliver, thus not perceived as adding substantially to their workload. The brevity of the intervention means it can be offered to every pregnant woman at every contact by a community midwife, or other health professionals such as GPs and obstetricians. It is possible that our intervention will produce a smaller effect than has been achieved by more intensive interventions [47–49], but these intensive interventions cannot be given to all pregnant women due to their high intensity and related costs. Our ambition is to have a modest but important impact on most of the 813,000 women giving birth in the UK each year, as well as those in other countries.

### Strengths and limitations

The study findings should be interpreted in light of its strengths and weakness. We have conducted a feasibility trial that recruited a small sample, not an effectiveness trial, and results should be interpreted as such. We did not record the antenatal consultations for either group and relied on self report of what happened. Whilst the follow up rates for assessments of weight before and after pregnancy were very high (89–90 %), the follow up rates for the questionnaire data were more modest, ranging between 62–74 % depending on the time This means the questionnaire data should be interpreted with some caution. Researchers taking follow up were not blinded to group allocation. Whilst several countries weigh women routinely there is no evidence that weighing alone is an effective weight management intervention for pregnant women and we know of no other study that has examined possibility of introducing regular weighing, feedback and setting and adjusting maximum weight targets by community midwives into routine antenatal care (or by other health professionals) or that has tested the intervention package we included here, so this study will make a unique contribution to the literature.

## Conclusions

In conclusion, this feasibility trial and qualitative study has shown that an intervention where pregnant women are routinely weighed, set weight gain limits, provided with feedback on their gestational weight gain and encouraged to weigh themselves weekly by community midwives throughout pregnancy is feasible to deliver within routine antenatal care. Using the lessons learnt here and with a greater emphasis in the intervention on women self monitoring their own weight gain. We now plan to conduct a phase III RCT to test the effectiveness of this intervention to prevent excessive gestational weight gain.

## Abbreviations

BMI: Body mass index
IOM: Institute of medicine
IMD: Index of multiple deprivation
UK: United Kingdom
NICE: National institute for health and care Excellence
RCTs: Randomised controlled trial
GP: General practitioner
